# Toxicokinetics of Metals in Terrestrial Invertebrates: Making Things Straight with the One-Compartment Principle

**DOI:** 10.1371/journal.pone.0108740

**Published:** 2014-09-30

**Authors:** Boris Skip, Agnieszka J. Bednarska, Ryszard Laskowski

**Affiliations:** 1 Institute of Environmental Sciences, Jagiellonian University, Kraków, Poland; 2 Institute of Nature Conservation, Polish Academy of Sciences, Kraków, Poland; 3 Department of Physical Chemistry and Environmental Chemistry, Chernivtsi National University, Chernivtsi, Ukraine; German Cancer Research Center, Germany

## Abstract

In this analysis, we first performed a critical review of one-compartment models used to describe metal toxicokinetics in invertebrates and found mathematical or conceptual errors in almost all published studies. In some publications, the models used do not represent the exact solution of the underlying one-compartment differential equations; others use unrealistic assumptions about constant background metal concentration and/or zero metal concentration in uncontaminated medium. Herein we present exact solutions of two differential-equation models, one describing simple two-stage toxicokinetics (metal toxicokinetic follows the experimental phases: the uptake phase and the decontamination phase) and another that can be applied for more complex three-stage patterns (toxicokinetic pattern does not follow two phases determined by an experimenter). Using two case studies for carabids exposed via food, based on previously published data, we discuss and compare our models to those originally used to analyze the data. Our conclusion is that when metal toxicokinetic follows a one-compartment model, the exact solution of a set of differential equations should be used. The proposed models allow assimilation and elimination rates to change between toxicokinetic stages, and the three-stage model is flexible enough to fit patterns that are more complex than the classic two-stage model can handle.

## Introduction

One of the major challenges in assessing potential effects of toxicants to organisms, to efficiently counteract their negative impacts on the environment, is explicitly predicting the internal active concentration of toxic chemicals in the body and/or target organs. Toxic effects estimated on the basis of internal body/tissue concentrations rather than on external exposure (e.g., concentration in food) are often far less variable among species, different chemicals with similar mode of action, and different environmental conditions [1]. Toxicokinetics translate an external concentration of a toxicant to an internal concentration over time. In the simplest form, toxicokinetic model includes the processes of assimilation and elimination of a toxicant, but more complex models may also include processes of biotransformation or internal distribution. In case of metals (which do not degrade like, e.g., pesticides) the simplistic toxicokinetic models are usually used for invertebrates. In such models, mathematical equations are fitted to experimental data on body burden as a function of time in an organism exposed to the contaminated medium. The models allow for estimating toxicant assimilation and elimination rates which can be used further for predictive simulations. Toxicant concentrations studied usually represent those found in the field although toxicokinetic parameters can be tested under different scenarios, allowing, for example, for determining relationships between concentration and uptake or elimination rates. Different time may be needed to initiate specific physiological processes of effective metal regulation in an organism, which may additionally change over the exposure period and differ among metals. Typically, it is assumed that for terrestrial invertebrates exposed via food ca. four-week period of feeding with metal-contaminated food (uptake phase) is long enough to reach an equilibrium body metal level ([2], [3], [4]), if such a level does exist for a particular metal and species. Estimation of assimilation rate in the presence of simultaneous elimination is improved significantly if the uptake is followed by decontamination phase at which animals are offered uncontaminated food [5].

A review of the literature on metal toxicokinetics in invertebrates yields a rather confusing picture. Toxicokinetic models differ substantially across publications. The result is that comparisons among species and metals are difficult (if not impossible), leading to the question of whether all of these models are sensible from a biological point of view and mathematically correct. The aim of this article is a critical evaluation of earlier publications on metal kinetics in invertebrates and the proposal of a set of models that can be easily implemented in toxicokinetic studies. The intent is for this analysis and the conclusions to facilitate further research on (metal) toxicokinetics and ease the choice of a proper model. Although the one-compartment principle is not restricted to terrestrial invertebrates, we focus here on case studies on carabids exposed via food only to avoid complexity connected with different exposure routes (e.g., exposure via food and skin in soil-dwelling organisms or via skin and gills in aquatic species).

Most authors studying metal toxicokinetics quote either Atkins [6] as the original source of the model(s) used (e.g., [7]) or refer to Janssen et al. [4], who based their model on Atkins [6] ([8], [9], [10]). Consequently, we start our critique by evaluating the applicability of these models to studies on metal toxicokinetics. For our purposes, we concentrate on classic compartment models, despite the recent paper by Argasiński et al. [11] in which the authors generally criticized the classic compartment models as purely phenomenological and not really describing processes underlying (metal) toxicokinetics. Thus, although we admit all the weaknesses of the traditional compartment models that Argasiński et al. [11] point out, we also acknowledge the practical fact that these models will still be used in the coming years as a convenient tool for describing metal kinetics in animals, at least phenomenologically. If so, we should ensure that the models are sensible, correct, and comparable with each other.

Toxicokinetics of metals can be theoretically described with a range of compartment models of varying complexity. The simplest one-compartment first-order model treats an animal as a ‘black box’ assimilating a chemical from the consumed food or other exposure medium (water, soil) at a rate *k_A_* and excreting (eliminating) it at a rate *k_E_*. More complicated models add more compartments; these may represent, for example, specific organs responsible for (temporary) storage of a chemical; e.g., Morgan and Morgan [12] found distinct differences in the distribution of various metals throughout the earthworm's body. Metal sequestration on a sub-cellular level has been summarized by Vijver et al. [13], who found it likely that differences in metal sequestration affect earthworm metal excretion. Further studies have indicated that different metal fractions may have their own specific uptake and elimination kinetics in the earthworm *Lumbricus rubellus* exposed via soil [14]. However, most studies on invertebrates indicate that metal toxicokinetics is sufficiently well described by the simplest one-compartment first-order model, which is widely used, although sometimes with minor modifications (e.g., [2], [4], [15]). Janssen et al. [4] formally tested five models, including two varieties of two-compartment and three varieties of one-compartment models. They evaluated cadmium kinetics in four species of soil arthropods and concluded that the one-compartment model with a fixed initial body concentration gave the best fit. On the other hand, the classic one-compartment model did not fit at all to the data on nickel toxicokinetics in the ground beetle *Pterostichus oblongopunctatus* [16], and Laskowski et al. [17] identified a number of studies in which two stages could be distinguished during metal uptake (i.e., when an animal is exposed to contaminated food).

Below we describe two simple models that we found to be reasonable from the perspective of animal physiology and present their exact mathematical solutions. Accompanying this analysis is a critical review of the models used in the published studies. If not stated otherwise, we assumed a typical toxicokinetic experiment consisting of two phases: the ‘uptake phase’ and the ‘decontamination phase’. During the uptake phase, an animal is exposed for a certain time to metal-contaminated food, and afterwards, at time *t_c_*, the food is changed to uncontaminated.

## The Models

The models presented below are the simplest one-compartment models that can satisfactorily describe most published data sets on metal toxicokinetics in terrestrial invertebrates. As mentioned above, the models probably do not capture the actual physiological and biochemical processes determining metal kinetics in an animal body but are simple descriptions to be used when no more detailed information is available. With this reservation, the parameters estimated from the models can be used to compare toxicokinetics among animal species, metals, and their different concentrations in medium (e.g., food, water, soil). Regardless of neglecting actual internal organismal processes, these models may tell us, for example, to what extent animals differ in their ability to control internal metal concentrations; whether assimilation and elimination rates of all metals are the same; and if they depend on metal concentration in food. Last but not least, estimating assimilation and elimination constants allow for calculating the equilibrium concentration of a metal in an animal body under specific environmental concentrations, information that may be crucial for assessing the risk of harmful effects in chronic exposures.

When defining each model, we start with differential equations, which are easy to understand and clearly show all of the important components. Such equations can be solved using common integration techniques, such as Euler or Laplace transforms [18]. The integration is out of the scope of this article because we do not want to burden a reader with formal math routines. Instead, we focus on the correctness of the equations used and point out the restrictions of their modifications and use. Throughout the article, we use the same set of symbols for specific model parameters (Table 1). For consistency, we apply the term ‘phase’ with respect to experimental phases (uptake or decontamination) and ‘stage’ for observed stages in toxicokinetics (which may but does not need to be equivalent to the experimental phases). Also, because different authors use different terms for processes connected with metal toxicokinetics, we had to define precisely those used in this work. In some papers, “uptake” and, respectively, “uptake constant” were used for the influx of metals into the animal body, expressed as a fraction of metal contained in food. Other authors used “assimilation” and “assimilation constant”, reserving the term “uptake” or “accumulation” for the total amount of metal actually entering animal body (e.g., milligrams of metal per gram body mass per day; i.e., *k_A_ • C_E_*). Because “assimilation” has a clear meaning in physiology and “uptake” does not, we use the former approach here. The term “uptake” here means only that an animal consumes contaminated food. The term “accumulation” references the amount of metal entering an animal body per unit mass per day (*k_A_ • C_E_*). For these reasons, we also encourage other researchers to follow this nomenclature.

**Table 1:** Symbols used in the article and the units of model parameters.

Symbol	Meaning [unit]
*t*	Time [days]
*t_c_*	Time of changing the food from contaminated to uncontaminated (exact time of the switch from the uptake phase to decontamination phase – defined by an experimenter) [days]
*t_s_*	Time when an animal ‘switches’ from high metal accumulation rate to low accumulation rate (in selected models only; defined by animal physiology; value estimated from the model) [days]
*C_I_*	Internal metal concentration in animal body (measured) [unit depends on the experimental setup and purpose, usually mg • kg^−1^ or mMol • kg^−1^]
*C_I0_*	Internal metal concentration in animal body at the start of the uptake phase, i.e., at *t* = 0 (measured) [unit as for *C_I_*]
*C_E_*	External metal concentration in food; additional index *u* can be used when relating to the uptake phase; additional index *d* can be used when relating to the decontamination phase (measured) [unit as for *C_I_*]
*C_Eu_*	External metal concentration in the uptake phase [unit as for *C_I_*]
*C_Ed_*	External metal concentration in the decontamination phase [unit as for *C_I_*]
*k_A_*	Assimilation rate constant; can be used with additional indices 1, 2,…, n if more than one assimilation constant is defined for different stages (estimated from the model) [day^−1^]
*k_E_*	Elimination rate constant; can be used with additional indices 1, 2,…, n if more than one elimination constant is defined for different stages (estimated from the model) [day^−1^]

Another issue requiring clarification is the units used in toxicokinetic models (cf. Table 1) because some confusion arises in the published literature. Certain inconsistencies are minor and stem simply from using either mass (e.g., [mg • kg^−1^]) or molar (e.g., [mM • kg^−1^]) units for expressing toxicant concentrations. As long as the use of concentration units is consistent in a model, the choice is mostly subjective. Nevertheless, in experiments comparing toxicokinetics of different metals, molar units would be preferred for obvious reasons: Metal concentrations are then expressed as an amount of ions/atoms entering the animal body. More problematic are the units used for toxicokinetics parameters. Some authors do not estimate the assimilation constant *k_A_* but rather estimate the accumulation rate (e.g., [2], [4]), which is the product of *k_A_* and *C_E_*. In such a case, the unit depends on how the concentration is expressed and can be, for example, [mg • kg^−1^ • day^−1^] or [mM • kg^−1^ • day^−1^]. The advantage of this approach is that the accumulation rate is easy to understand because it indicates directly how much of a toxicant enters the animal body per unit time. In a more traditional approach, when both *k_A_* and *k_E_* are estimated, different units are used for *k_A_*. However, in this case, the actual unit also should be derived from appropriate equations: For both *k_A_* and *k_E_*, this derivation should yield the inverse of the time unit, most commonly [day^−1^]. The rather strange ‘unit’ [g_food_ • g_body_^−1^ • day^−1^] used sometimes for *k_A_* is supposed to express the fact that this parameter describes the rate of toxicant assimilation per body mass from a unit of food. However, such a unit cannot be derived from the equation describing the one-compartment model and conflicts with the understanding of the two toxicokinetic constants *k_A_* and *k_E_*. First, the actual mass unit here is [g], whether it is a gram of food or body, so the units cancel each other out in the equation, leaving only [day^−1^]. Second, because *k_A_/k_E_* is a bioaccumulation factor, which is dimensionless by definition, both *k_A_* and *k_E_* must be expressed in the same units so that those units cancel each other out, as well. Note also that if the accumulation rate, which is *C_E_* • *k_A_*, is expressed in [mg • kg^−1^ • day^−1^], *C_E_* being [mg • kg^−1^], then *k_A_* must be expressed in [day^−1^]. Thus, the proper unit for both *k_A_* and *k_E_* is [day^−1^].

### The one-compartment model with two stages

In this section, we assume that metal toxicokinetic follows the simple one-compartment model with two phases determined by an experimenter: the uptake phase and the decontamination phase. The observed toxicokinetic pattern follows the experimental phases, resulting in two stages. The simplest ‘black box’ one-compartment model is described by the following equation:
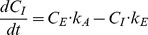
(1)


Eq. 1 is the general model describing changes in body metal concentration in time at a specific external concentration of the metal (*C_E_*) in food, without specifying whether it is the ‘uptake phase’ or the ‘decontamination phase’. The model has two solutions, depending on initial concentration in the animal body, C*_I_*_0_, at the beginning of exposure to contaminated food (*t* = 0):
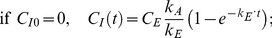
(2)

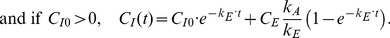
(3)Please note that Eq. 2 is correct only for chemicals that do not occur naturally in the environment, so that their concentration in the animal body before exposure can indeed be assumed to be zero. Eq. 2 can thus be used, for example, to describe the toxicokinetics of pesticides or pharmaceuticals but not metals, which are always present at concentrations greater than zero. In the latter case, only Eq. 3 is correct. However, Janssen et al. [4] proposed a slightly different model, which they used to describe cadmium kinetics in invertebrates, assuming the existence of some ‘inexchangeable’ metal body burden *C_I_*_0_ instead of *C_I_*_0_·*exp(−k_E_ • t)* as it follows from the exact solution (Eq. 3):
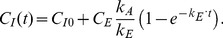
(4)


Because in terms of the fit to data sets, the model (Eq. 4) appeared to be the best among the five tested by Jansen et al. [4], and indeed described the observed patterns well, several authors adopted for in later studies (e.g., [2], [3], [8], [10], [19], [20], [21], [22]). The idea of adding some non-zero constant to the model, namely a constant ‘background’ body concentration, seemed reasonable because all organisms do maintain certain concentrations of all chemical elements. Moreover, as Janssen et al. [4] noted, omitting this term results in a decrease in the modeled metal concentration down to zero during the decontamination phase when the simple decay function is used, which cannot be correct. Apparently noticing this problem, Nahmani et al. [23] distinguished between essential metals, which must have certain non-zero levels in organisms, and non-essential metals which, according to their reasoning, can reach zero concentration as an effect of decontamination. Consequently, they used Eq. 4 to describe the toxicokinetics of essential metals and Eq. 3 for non-essential ones. However, none of these approaches is correct. First, Eq. 4 is not the exact solution of the underlying differential equation. Second, there are no good physiological grounds for the assumption that ‘non-essential’ and ‘essential’ metals are regulated by substantially different mechanisms (in fact, it can be just the opposite; e.g., zinc and cadmium can be fixed by the same granules and proteins). Third, we are still not sure which metals can be qualified as ‘non-essential’, as proved by a relatively recent discovery of a Cd-based enzyme in a marine diatom [24]. Heugens et al. [25], who studied accumulation kinetics of cadmium in *Daphnia magna*, used the correct equation (Eq. 3) without any unnecessary assumptions, as did Tsai et al. [26] in their study on Cu accumulation in tilapia (*Oreochromis mossambicus*).

The problem of metal concentrations decreasing to zero during the decontamination phase stems from a fundamental error in describing the process. In most papers on metal toxicokinetics in animals, a simple exponential decay model has been assumed for this phase:
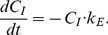
(5)


Although Eq. 5 and the corresponding exact solution indeed describe well many decay-like processes, including decontamination of pesticides, they do not make sense in the case of metals. In contrast to human-made chemicals, such as many pesticides, metals are always present in the environment at certain background concentrations. For some metals, these natural concentrations are actually not that low; for example, Zn concentrations in uncontaminated soils can range between ca. 10 and 300 mg kg^−1^ [27]. Thus, even during the decontamination phase of a typical toxicokinetic experiment, animals still consume food with non-zero metal concentrations and/or live in a medium containing some background metal levels. Consequently, also in this phase, metal toxicokinetics should be described by the equation analogous to Eq. 1, which results in a set of two similar equations that differ only in external metal concentrations because the concentration in the uptake phase (*C_Eu_*) is usually much higher than the background concentration in the decontamination phase (*C_Ed_*):
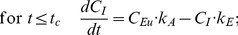
(6)

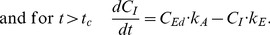
(7)Assuming *C_I_*_0_>0, the integration of Eqs. 6 and 7 results in the following models, similar to Tsai et al. [26]:
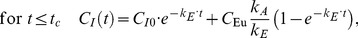
(8)

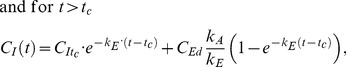
(9)where
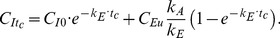
(10)


With such a mechanistically defined one-compartment toxicokinetic model, there is no need to make biologically unrealistic assumptions that normal background metal concentration in a body is fixed and does not depend on external conditions [4] or that non-essential metals are eliminated to null [28]. For both the essential and non-essential metals, an equilibrium concentration is eventually reached during decontamination which is the final concentration expected at a specific background metal content in a food or medium. This final concentration can be calculated assuming t→∞ for Eq. 9:
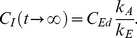
(11)


Equations 3, 8, and 9 are the exact solutions of the set of differential equations describing the one-compartment kinetics of those chemicals that never reach zero concentration in the environment and organisms, as it follows from Eq. 11. The model is also sensible from a biological perspective because organisms indeed always consume some amount of metals with food and live in environments with certain concentrations of metals. It is also well known that if concentrations of essential metals in a food/medium drop below organism requirements, the result can be serious health problems, as is the case of anemia arising from an insufficient iron supply.

While the model is mathematically and biologically correct, there is yet one more unknown about it: Although we call *k_A_* the ‘assimilation rate constant’ and *k_E_* the ‘elimination rate constant’, we actually know that these parameters are constant only for certain conditions, such as specific metal concentration or particular and constant temperature [26], [29]. For example, Tsai et al. [26] revealed that Cu toxicokinetics in tilapia (*Oreochromis mossambicus*) depend on concentration and time, suggesting that Cu burdens were under physiological control. The model Tsai et al. [26] used tended to overestimate Cu concentrations in some organs under extended exposure periods. The authors assumed that this happened because the model does not consider possible changes in the values of uptake and depuration rates and questioned the usefulness of the classic toxicokinetics model to illustrate metal accumulation, especially under prolonged exposure. Thus, when an animal is switched from the uptake phase to the decontamination phase, *k_A_* and *k_E_* may also change. Kramarz [2], [3] had already noted the need to estimate elimination rates separately for each phase, and the estimated *k_E_* values indeed differed between the uptake and elimination phases. It may be thus advisable to treat the assimilation and elimination constants with caution and plan toxicokinetic experiments in such a way that each phase has sampling dense enough to allow for separate estimation of the model parameters: *k_A1_* and *k_E1_* for the uptake phase, and k*_A_*_2_ and *k_E2_* for the decontamination phase. Different rate constants for the uptake and decontamination phases may indicate complexity that goes beyond the first-order kinetics. This possibility is, however, out of the scope of the present paper.

### The one-compartment model with three stages

Although in many cases, the one-compartment model with two stages, equivalent to the uptake and decontamination phases, describes metal toxicokinetics satisfactorily, a few recent studies indicate that (certain?) metals (at some concentrations?) do not always follow this pattern [16], [20], [30], [31]. These suggestions led Laskowski et al. [17] to propose a modified model with three stages. Stages I and III are similar to those used to describe metal toxicokinetics in the classic two-stage model; that is, the initial uptake and final elimination of the metal. Thus, in stage I, an animal accumulates a metal from contaminated food/environment as described by Eq. 6 (and the respective integrated form, Eq. 8), and in stage III, after moving the animal to uncontaminated food/medium (*t*>*t_c_*), the excess of the metal is eliminated from the body and eventually reaches the pre-exposure level, as indicated by Eq. 9. However, in between stages I and III, an additional stage in metal kinetics can be distinguished: metal concentration in the animal body, after reaching a maximum at *t* = *t_s_* (‘switch time’), starts decreasing even if the animal is still exposed to the same metal-contaminated food. The concentration drops to some semi-steady level, which is higher than in uncontaminated animals but substantially lower than the maximum reached in stage I. The reasons for such kinetics are unknown, but at least three mechanisms can be involved. First, after a certain concentration threshold, animals can deliberately increase the elimination rate *k_E_* to avoid further intoxication. Second, after a high accumulation of a metal in stage I, animals can decrease the metal assimilation rate *k_A_*, either because a high uptake rate is no longer necessary (in cases of some essential metals that can be limiting in uncontaminated environments) or can lead to toxic concentrations. Third, the two mechanisms can work together towards better, more efficient control over internal metal concentrations. Argasinski et al. [11] proposed that this regulation does not require any sophisticated mechanisms fine-tuning the assimilation and elimination rates to actual metal concentrations in food but can result simply from direct toxicity of metal ions to gut epithelial cells. The model they proposed seems plausible but is neither well tested yet nor can be fitted to actual data. On the other hand, the model published by Laskowski et al. [17] is a purely phenomenological description of the pattern, without a well-established underlying mechanism. Because we do need a model that can handle such ‘non-standard’ metal kinetics, we propose here a formal three-stage model based on the following assumptions:

1. The data on toxicokinetics originate from a classic two-phase experiment.
2. Metal toxicokinetic reveals three stages: two in the uptake phase (stage I, characterized by a fast increase in internal concentration, and stage II, starting with a decrease in concentration followed by a semi-steady concentration), and one in the decontamination phase, when internal metal concentration decreases to the pre-exposure concentration (being at the same time an equilibrium concentration with the uncontaminated food/environment).
3. Assimilation and elimination rates are not constant throughout the exposure to both the contaminated and the uncontaminated food but change among the three stages.

Without detailed physiological studies, we cannot tell whether internal metal concentration is regulated through changing *k_A_*, *k_E_*, or both. Hence, in the mathematical formulation of the model, we allow both parameters to change between the stages but freeze the constants within each stage. For the purpose of the three-stage model, we need to introduce the time *t_s_* when an animal ‘switches’ from stage I to stage II, which is estimated from the model; alternatively, the time to reaching maximum concentration can be used as *t_s_* if sufficiently dense sampling is done. Indices 1, 2, and 3 next to assimilation and elimination rates denote respective toxicokinetic stages. The set of differential equations looks as follows:
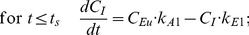
(12)

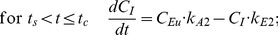
(13)

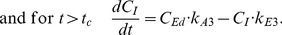
(14)


As in all models for metal toxicokinetics, we assume positive, non-zero initial conditions, that is C_I*0*_>0. Integration of Eqs. 12–14 then results in the following models:

Phase I, stage I (*t*≤*t_s_*),
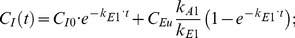
(15)


Phase I, stage II (*t_s_<t*≤*t_c_*),
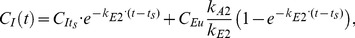
(16)where 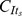 is the initial concentration for the second stage, calculated from Eq. 15 for *t = t_s_*:
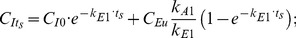
(17)And Phase II, stage III (*t*>*t_d_*),
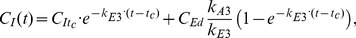
(18)where 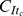 is the initial concentration for the third stage, calculated from Eq. 17 *t = t_c_*:
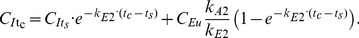
(19)


In the case studies presented below, the respective models were fitted to the data from two published articles: Kramarz [2], exemplifying a clear two-stage toxicokinetics, and Laskowski et al. [17], in which three distinct stages could be seen. The models were fitted with MatLab R2012B, using the program code with the «fit()» function and corresponding options for fit: nonlinear least squares method for minimization of SSE, and the ‘trust-region’ algorithm for bounding the scanned variable intervals, specifying the optimization start point for estimated parameters and specifying low bound for scan. The minimization procedure and the results of parameter estimation appeared to be sensitive simultaneously to the initial value (startpoint) of the switch point (*t_s_*) and the lower bounds of *t_s_*. As a result, the fit was done in cycles from the lowest bound value to *t_c_* in the interval [0; *t_c_*]. The lowest bound is the minimal value of estimated parameters that can be considered during the estimation. The initial point is the start point for the estimation procedure. The best fit was chosen based on the lowest value of collected SSE in a two-dimensional array. If the cycle procedure was not successful (i.e., at least one of the estimated values reached one of the bounds), the single fitting was used with zero as the initial value and the start point for *t_s_*.

## Case Studies

### The one-compartment model with two stages

As a case study, the data from a previously published experiment on Cd and Zn toxicokinetics in the ground beetle *Poecilus cupreus* were used [2]. For the purpose of this article, we used data from only the Cd treatment. Summarizing the experiment briefly, adult beetles originating from a laboratory culture were individually fed housefly larvae (*Musca domestica* L.) reared on artificial medium contaminated with Cd at 50 mg kg^−1^ dry weight of food. The animals were fed contaminated larvae for 90 days (uptake phase) and afterwards transferred to control food (decontamination phase). Twenty beetles were analyzed before starting the experiment (day 0) to determine initial Cd body concentration (*C_I_*_0_). During the uptake phase, six randomly chosen beetles were sampled after 2, 6, and 13 days of exposure and weekly thereafter. After being transferred to uncontaminated food, six beetles were sampled after 92, 96, 100, 107, 114, and 121 days. To void their gut, the beetles were starved for 24 h and then frozen for Cd analyses. The beetles and their diet were analyzed for Cd concentration with a graphite furnace atomic absorption spectrometry (AAS). The actual Cd concentration (per dry mass) in Cd-contaminated housefly larvae was 155±2.2 mg kg^−1^ (mean±SE). Because Cd concentration in control housefly larvae was reported as being below the detection level, the value of 0.92±2.2 mg kg^−1^ was taken from Maryański et al. [32].

Originally the data were analyzed using a one-compartment two-stage model [2], but the elimination constants were allowed to differ between the experimental phases. Similarly to Jansen et al. [4], Kramarz [2] did not use the exact solution of the differential model in that study. Below, we compare the results of fitting to the same data the model by Janssen at al. [4], the one by Kramarz [2], and two versions of the exact solution: one with the same assimilation and elimination rates in both phases of the experiment, and a second allowing the assimilation and elimination rates to differ between the phases.

### The one-compartment model with three stages

To illustrate the case in which three stages in metal toxicokinetics can be clearly distinguished, we used data from the previously published experiment on nickel toxicokinetics in the ground beetle *Pterostichus oblongopunctatus*, which are exactly the same data used by Laskowski et al. [17]. The beetles were kept individually in 30-ml plastic vials filled ¾ with moist peat (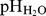 4.5–5.0, 80% WHC) at 20°C and fed *ad libitum* with artificial food made of ground mealworms mixed with ground apple. In a 96-day experiment, the animals were exposed to nickel-contaminated food (nominal concentration 2500 mg per kg dry weight) for 64 days (uptake phase) and afterwards transferred to uncontaminated food (decontamination phase). Twenty-one beetles were analyzed before starting the experiment (day 0) to determine initial Ni body concentration (*C_I_*_0_). During the uptake phase, four beetles were sampled after 2, 4, 6, 8, 16, 32, and 64 days of exposure. After 64 days, the remaining beetles were transferred to clean food, and four beetles were sampled after 66, 68, 80, and 96 days. The sampled beetles were allowed to void their guts for 48 hours and then were frozen at −20°C. The beetles and dry food were analyzed for nickel concentration with a graphite furnace AAS. The actual Ni concentrations in uncontaminated and contaminated food were 5.95±2.25 (mean±SD) and 2525±76 mg kg^−1^, respectively. For more details see [17].

The data were originally analyzed with two different models to accommodate the unexpected three-stage behavior [11], [33], but neither of them represented the exact solution of the set of differential equations described above (Eqs. 12–14). The three-stage model used by Laskowski et al. [17] allowed for an estimated early breakpoint (t_s_), after which metal concentration decreased even in the uptake phase, and for a single assimilation rate constant (*k_A_*) and two elimination rate constants: one during the initial phase of quick concentration increase (*k_E1_*) and another one (*k_E2_*) after the breakpoint. The model also contained two additional empirically derived parameters: the asymptote A and the final concentration after depuration *C_f_*. Theoretically, with more densely sampled animals, the model could also be tested for separate assimilation rates, but the available data did not allow for that. Here we use these data to compare the model originally estimated by Laskowski et al. [17], later called 3L, against two versions of the exact-solution model (Eqs. 15–19), the first with one *k_A_* throughout the experiment and only *k_E_* changing between the first and second stages (*k_E1_*, *k_E2_*) of the observed toxicokinetic pattern, as in the model by Laskowski et al. [17], called 3E1; the second with separate assimilation and elimination rates estimated for each toxicokinetic stage (*k_A1_*, *k_E1_*, *k_A2_*, *k_E2_*, *k_A3_*, *k_E3_*), called 3E2. When estimating model parameters, the following data were used: *C_I_*_0_ = 0.71 mg kg^−1^, *C_Eu_* = 2525 mg kg^−1^, *C_Ed_* = 5.95 mg kg^−1^, and *t_c_* = 64 days.

## Results and Discussion

### The one-compartment model with two stages

**Figure 1:**
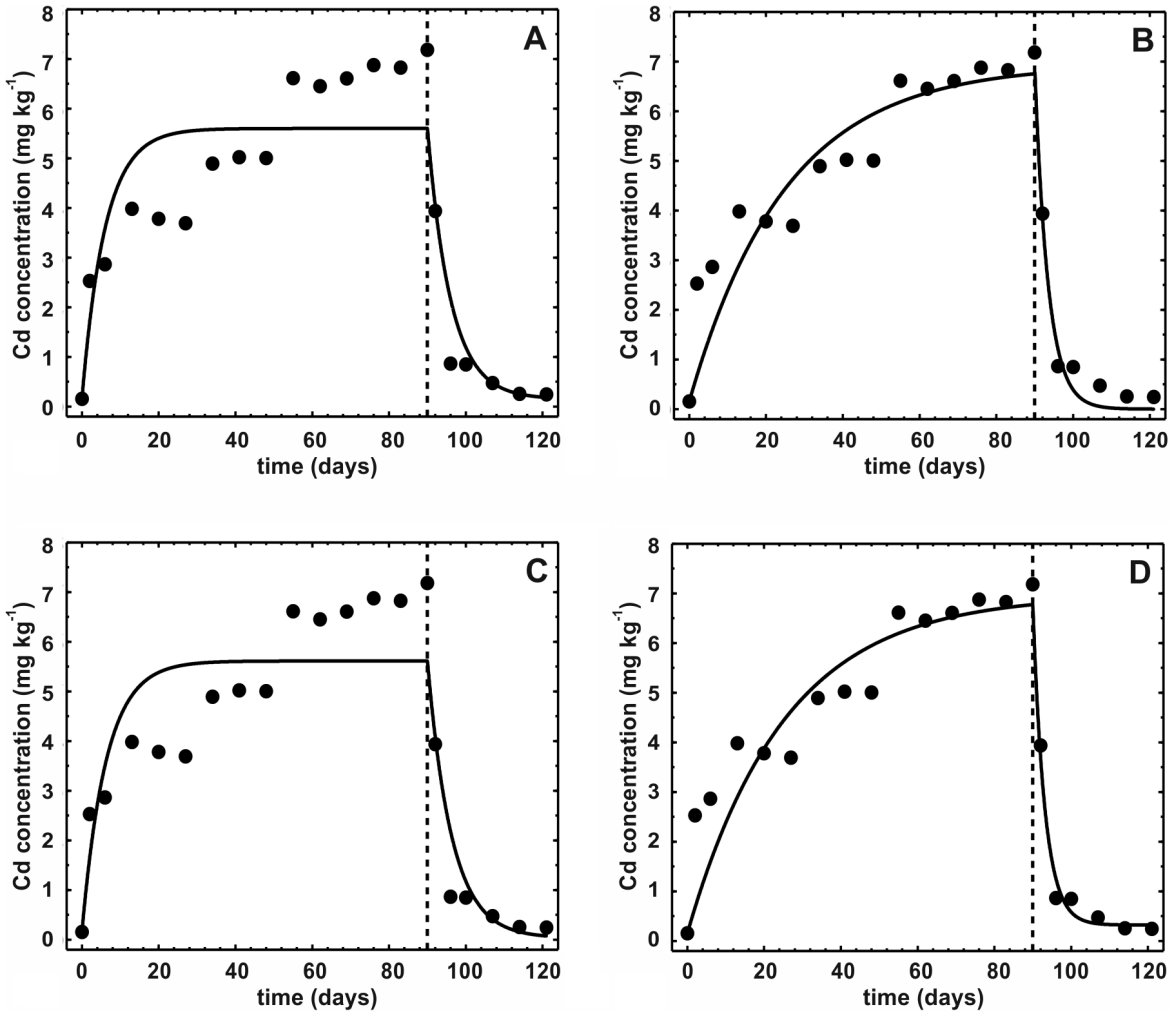
Two-stage toxicokinetic models. Two-stage toxicokinetic models fitted to mean Cd concentrations in the ground beetle *Poecilus cupreus* (data from Kramarz [2]): a) model by Janssen at al. [4] with constant *C_I0_* = 0.15 mg kg^−1^ and *C_Eu_* = 154.6 mg kg^−1^; b) model originally used by Kramarz [2] with *C_I0_* = 0.15 mg kg^−1^ and *C_Eu_* = 154.6 mg kg^−1^; c) exact-solution model (Eqs. 8 and 9) with the same uptake and elimination rates in both phases of the experiment, *C_Eu_* = 154.6 mg kg^−1^ and *C_Ed_* = 0.92 mg kg^−1^; d) exact-solution model with different uptake and elimination rates in the first and second phases of the experiment, *C_Eu_* = 154.6 mg kg^−1^ and *C_Ed_* = 0.92 mg kg^−1^. Vertical broken line indicates the day of changing to uncontaminated food (*t_c_*).

Comparison of the original model by Jansen et al. [4] (i.e., with constant *C_I_*_0_, and one *k_E_* throughout both phases of the experiment); the one used by Kramarz [2] (i.e., the model by Jansen et al. [4] modified to allow the *k_E_* to differ between the uptake and the decontamination phases – *k_E1_* and *k_E2_*); and two versions of the exact solution of the differential equations 8 and 9: with (1) common *k_A_* and *k_E_* values for both phases of the experiment and (2) separate assimilation and elimination rates for each phase (*k_A1_, k_E1_, k_A2_, k_E2_*) showed that the two models allowing the constants to differ between the phases gave a clearly better fit (Table 2, Fig. 1). This outcome confirms that the elimination and/or assimilation rates indeed differ between the phases, as postulated by Kramarz [2]. The finding seems reasonable from the physiological point of view because animals should adjust accumulation rates of metals depending on their instantaneous external and internal concentrations (cf. [11]). There is no biological reason why *k_A_* and *k_E_* should be maintained constant throughout the uptake and decontamination phases, regardless of sometimes drastically different metal concentrations in food. The models assuming the same assimilation and elimination rates in both phases of a typical toxicokinetic experiment thus should be rejected in future studies.

**Table 2:** Comparison of the four two-stage toxicokinetic models fitted to mean Cd concentrations in *Poecilus cupreus* [2]; 95% confidence intervals in brackets.

Parameter	Estimated value			
	Model by Janssen at al. [4]	Model by Kramarz [2]	Exact solution model, one *k_A_* and *k_E_*	Exact solution model, *k_A1_*, *k_A2_*, *k_E1_*, *k_E2_*
k_A1_	0.0058* (0.0029–0.0088)	0.0018* (0.0013–0.0024)	0.0057 (0.0029–0.0084)	0.0018 (0.0012–0.0024)
k_E1_	0.1655 (0.0814–0.2500)	0.0400 (0.0210–0.0590)	0.1565 (0.0802–0.2328)	0.0396 (0.0211–0.0580)
k_A2_				0.1115 (−0.2259–0.449)
k_E2_		0.287 (0.0045–0.5694)		0.321 (0.1149–0.5267)
R^2^	0.846	0.940	0.847	0.935
R^2^_adj_ #	0.837	0.924	0.839	0.914
P‡	<0.0001	<0.001	<0.001	<0.0001
BAF&	0.035	0.045	0.036	0.045

Please refer to Table 1 for symbols description.

*- calculated value from estimated parameter a (metal accumulation rate) for the corresponding model.

# - R^2^ adjusted for degrees of freedom.

& - bioaccumulation factor (BAF) calculated based on assimilation and elimination constants for the first stage of the one-compartment models (*k_A1_/k_E1_*).

‡ - p value for the model.

The exact solution with separate assimilation and elimination rates for the uptake and decontamination phases gave almost identical toxicokinetic parameters and R^2^_adj_ as those obtained by Kramarz [2], except for *k_A2_*, which was not estimated by Kramarz (Table 2). Although it thus may seem that it does not really matter which model is used, we recommend using the exact solution for at least three reasons. First, because it is the mathematically correct solution of a set of differential equations describing one-compartment kinetics. Second, because it does not require unrealistic assumptions about constant body concentration of a metal in animals fed uncontaminated food or zero concentration in uncontaminated food. Third, because even if in this particular case the parameters estimated with both methods were very similar, that is not necessarily always the case: For example, if the background concentration of a metal is high, then the difference between actual final body concentration and zero (as assumed by the simple decay model) may be substantial. Graphically, this effect can be seen even for cadmium (Fig. 1): Although the graphs depicting the model by Kramarz [2] and the exact solution with separate toxicokinetic constants in each phase look almost identical, the latter describes the decontamination phase better because it does not force the concentration drop to zero (as is the case when the simple exponential decay function is used for the decontamination phase).

### The one-compartment model with three stages

**Figure 2:**
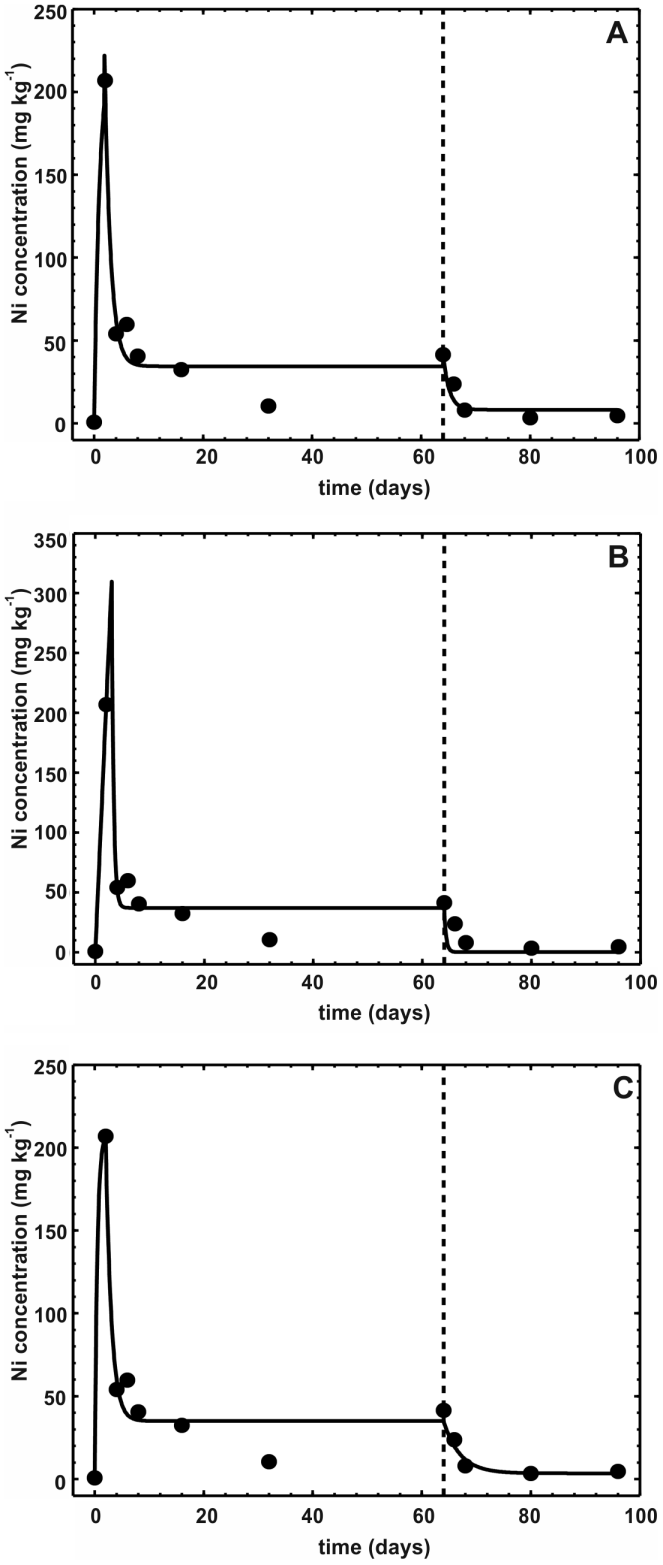
Three-stage toxicokinetic models. Three-stage toxicokinetic models fitted to geomean Ni concentrations in the ground beetle *Pterostichus oblongopunctatus*: a) model by Laskowski et al. [17]; b) exact-solution model, case scenario with one uptake rate and two different elimination rates; c) exact-solution model with all assimilation and elimination rates stage-specific (Eqs. 15–19). Vertical broken line indicates the day of changing to uncontaminated food (*t_c_*).

Comparison of the three versions of the three-stage model revealed almost identical fits in terms of both graphical representations (Fig. 2) and statistics: R^2^ was marginally higher for model 3E2 (0.966 vs. 0.963 for 3L vs. 0.95 for 3E1), but the high number of estimated parameters penalized the model in terms of the lowest, albeit also marginally, R^2^_adj_ (Table 3). One problem with the three-stage models is that with a regular toxicokinetic experiment, with rather few sampling points in time, the models easily appear overparameterized. This factor may make fitting the model to the data impossible and/or make the confidence intervals around the estimated parameters too broad to reach any conclusions about their significance or the significance of differences between the parameters in the three stages. The latter happened in our case study, even if the graphical representation of the models (Fig. 2) shows a very good fit to the data and all models were highly significant (p<0.0001, Table 3). One conclusion stemming from these results is that whenever a three-stage toxicokinetics is expected, the concentrations of metals in animals have to be measured much more frequently than in a typical experiment.

**Table 3:** Comparison of the three three-stage models of the complex nickel toxicokinetics in *Pterostichus oblongopunctatus* [17].

Parameter	Estimated values		
	Model by Laskowski et al. [17]	Exact solution model, *k_A_*, *k_E1_*, *k_E2_*	Exact solution model, *separate assimilation and elimination rates for each stage*
	Model 3L	Model 3E1	Model 3E2
k_A1_	0.091 (−2.6·10^7^, 2.6·10^7^)	0.0408 (−1.67, 1.751)	0.0751 (−1.2·10^7^, 1.2·10^7^)
k_E1_	1.022 (−1·10^8^, 1·10^8^)	10^−5^ (−41.64, 41.64)	0.6583 (−1.5·10^8^, 1.5·10^8^)
k_A2_			0.0132 (−0.0034, 0.0298)
k_E2_	0.8917 (−2.328, 4.111)	2.781 (−113.6, 119.1)	0.9497 (0.0054, 1.894)
k_A3_			0.1916 (−1.795, 2.178)
k_E3_			0.3311 (−0.7231, 1.385)
A†	34.43 (12.54, 56.31)		
C_f_ &	8.22 (−13.62, 30.06)		
t_s_	1.87 (−3.5·10^8^, 3.5·10^8^)	3.004 (−41.15, 47.16)	1.978 (−9.9·10^7^, 9.9·10^7^)
R^2^	0.963	0.9501	0.966
R^2^_adj_ #	0.932	0.924	0.923
P‡	<0.0001	<0.0001	<0.0001

Parameters have been estimated based on geomean Ni concentrations; 95% confidence intervals in brackets. Please refer to Table 1 for symbol description.

† - Asymptote – the semi-steady Ni concentration reached in the second stage of the toxicokinetics (cf. [17]).

& - Final concentration – the ultimate concentration reached during the decontamination phase (cf. [17]).

# - R^2^ adjusted for degrees of freedom.

‡ - p value for the model.

Although all three models gave very good fit to the data (in all cases, at least 95% of the total temporal variance in Ni body concentrations was explained), the estimated parameters differed vastly. Because each model contained a different set of parameters, not all can be compared. Among those present in all models, the estimated elimination rates especially differed substantially (Table 3). The clear benefit of model 3E2, if one accepts the broad confidence intervals around the estimated parameters, is that in contrast to the remaining two models, the meaning of the parameters is straightforward. As can be seen from Table 3 and Figure 2, when the beetles were exposed to Ni-contaminated food, a very fast increase in Ni body concentration was observed, with *k_A1_* = 0.0751 day^−1^ and *k_E1_* = 0.6583 day^−1^. However, after two days of exposure, the assimilation rate dropped substantially (*k_A2_* = 0.0132 day^−1^) with simultaneously increasing elimination efficiency (*k_E2_* = 0.9497 day^−1^). This outcome resulted in a fast decrease in body Ni concentration to a semi-steady concentration. The semi-steady concentration was estimated by Laskowski et al. [17] explicitly as the asymptote *A* = 34.4 mg kg^−1^. The exact-solution model does not allow for direct estimation of this asymptotic concentration, but it can be calculated as *C_Eu_* • *k_A2_*/*k_E2_* for model 3E2 or *C_Eu_* • *k_A_*/*k_E2_* for model 3E1, resulting in 35.1 mg kg^−1^ and 37.0 mg kg^−1^, respectively. Similarly, the final ultimate concentrations in the beetles after decontamination can be compared between the models. In model 3L, it was also estimated explicitly and equaled 8.22 mg kg^−1^. The value calculated for model 3E1 as *C_Ed_* • *k_A_*/*k_E2_* was 0.087 mg kg^−1^, and for 3E2, as *C_Ed_* • *k_A3_*/*k_E3_*, it was 3.44 mg kg^−1^. Assuming that after a long decontamination, the beetles would reach a Ni concentration similar to that from before the exposure, which was *C_I_*_0_ = 0.71 mg kg^−1^, the concentration estimated by model 3E2 is clearly the closest to this value. In addition, model 3E2 graphically fits the last two data points better than the remaining two.

## Conclusions

We showed that whenever metal toxicokinetics is described with the one-compartment model, the exact solution of a set of differential equations may and should be used. The exact solution does not allow for unrealistic assumptions about constant concentrations of metals in animals exposed to uncontaminated food or zero metal concentrations in uncontaminated food. Instead, actual concentrations measured in food in both the uptake and the decontamination phases are to be used. The model also allows the assimilation and elimination rates to change between the toxicokinetics stages. The additional benefit of using this approach is that a theoretical ultimate body metal concentration can be calculated for any external concentration, including uncontaminated food or medium.

The three-stage model based on an exact solution of the differential equations has proved flexible enough to fit the actually observed pattern and a complexity greater than the classic two-stage model can handle. Although with available data we still cannot tell whether the observed regulation of metal concentrations results from changes in assimilation efficiency of the intestinal epithelium or rather from an increased elimination rate (or a combination of both; cf. [17]), our work indicates directions for further research and methods for analyzing complex toxicokinetic patterns.
